# Health care service utilization among elderly in rural setting of Gandaki province, Nepal: a mixed method study

**DOI:** 10.3389/frhs.2024.1321020

**Published:** 2024-09-25

**Authors:** Kamal Poudel, Dinesh Kumar Malla, Kanchan Thapa

**Affiliations:** ^1^ Public Health Graduate, Shree Medical and Technical College, Purbanchal University, Chitwan, Nepal; ^2^ Department of Population Studies, Birendra Multiple Campus, Bharatpur, Chitwan, Nepal; ^3^ Department of Health and Social Research, Noble Shivapuri Research Institute, Budhanilkantha, Kathmandu, Nepal

**Keywords:** health service utilisation, elderly, mixed method analysis, Nepal, health service accesibility

## Abstract

**Introduction:**

Globally, one in every six people will be elderly by 2030. In Nepal, there has been a notable rise in the aging and elderly. Addressing the healthcare needs of them is crucial. Despite the different efforts to advocate for healthy aging, various factors continue to limit this process. This paper aims to explore the utilization of healthcare services among the elderly population and uncover influences on the ability to access these services.

**Method:**

A mixed-method community-based study was conducted in Bihadi Rural Municipality of Parbat, Nepal. The quantitative segment involved interviews with 355 individuals aged ≥60 years, while 18 respondents were enlisted for in-depth interviews. We used descriptive statistics, chi-square test, and logistic regression in quantitative analysis. Similarly, content and thematic analysis were performed in the qualitative component.

**Results:**

This study reported that health service utilization among the respondents was 65.4%. Among the factors ethnicity (OR 3.728, 95% CI 1.062–15.887), not good health status (OR 2.943, 95% CI 1.15–8.046), bus as means of transportation (OR 8.397, 95% CI 1.587–55.091) had higher odds whereas government hospital (OR 0.046, 95% CI 0.009–0.193), not always available health staffs (OR 0.375, 95% CI 0.147–0.931), not sufficient medicine (OR 0.372, 95% CI 0.143–0.924), not available medicine (OR 0.014, 95% CI 0.002–0.068) had lower odds for health service utilization. Other factors identified from qualitative components include long waiting times, insufficient medicine, lack of trained health personnel, financial capacity, low utilization of health insurance, distance, and support from family members.

**Conclusions:**

Nonetheless, a portion of the elderly remained excluded from mainstream of healthcare services. A combination of social, healthcare-related, and individual factors influences the utilization of healthcare services. To ensure elderly-friendly services, prioritize geriatric care training, secure medication availability, and establish a dedicated health insurance program for them. In the current federal context, localizing evidence-based, innovative strategies to address the healthcare needs of the elderly is crucial.

## 1 Introduction

Globally, the aging population is rapidly expanding, with approximately one-sixth of the world population expected to be 60 years and over by 2030, doubling the number from 2020. Similarly, the population of 80 or older will be tripled by 2050, reaching 426 million (1). The South Asian region is expected to rise in the elderly population, reaching 13.7% by 2030 and 20.3% by 2050, up from 9.8% in 2017 (2). Nepal is experiencing a rapid increase in its aging population, with the most recent data showing that there are 2,977,318 individuals (10.20%) of total population aged ≥60 years in the country according to the 2021 census (3). Over the past ten years, the percentage of senior individuals has climbed by 38%. The Terai Region has a somewhat lower percentage of elderly residents than Mountain and Hill. The proportion of senior individuals is greater in the provinces of Gandaki, Koshi, and Bagmati compared to the national average. Conversely, the provinces of Madhesh, Lumbini, Sudurpashchim, and Karnali have smaller percentages of senior citizens (4). This aligns with the global trend of a significant increase in the elderly population over the past two decades, including in Nepal (5).

In Nepal, the elderly population is expanding at a rate of 3.77%, significantly higher than the overall population growth rate of 1.35%, due to lower birth rates and increased life expectancy (6). According to the Nepal Senior Citizen Act 2063, elderly or senior citizens are those people who are 60 years or above (7). As of 2021, Nepal's total population is 29,192,480, with Gandaki province accounting for 8.49% (8).

The concerns and needs of senior citizens should be integrated into the core of the development agenda (9). Nepal has lacked focus on geriatric care, with many elderly individuals in rural areas seeking medical attention only when they fall ill or relying on self-medication. The healthcare service coverage index in Nepal is relatively low at approximately 46 (with a higher rank being above 80) and expanding Universal Health Care (UHC) coverage is a significant challenge (10). Remote healthcare facilities exhibit substandard quality and limited institutional capacity, indicating unequal emphasis within the healthcare sector (11).

Challenges associated with aging emerge when physical and mental changes impede an individual's daily tasks. With longer lifespans, chronic illnesses, common in old age, are the leading causes of disability and need for daily assistance. Tackling these challenges demands a holistic approach involving multiple sectors such as income security, healthcare, housing, transportation, recreation, and basic activities (12).

In previous study which was done in Estonia shows that elderly with chronic illness have more chances to utilize health care service (13). In another study from Butwal of Nepal, 84.4% of older people attended health facilities, with most managing chronic diseases and taking daily medication. Moreover, 84.9% of them reported good health (14). The results indicate that due to geographical constraints and limited transportation, many elderly people struggle to access health services. The issue is especially pronounced among poor older adults, who face high care costs and a lack of government healthcare coverage, posing significant financial risks. Despite the efforts to promote elderly welfare, effective implementation of national plans, policies, and global commitments remains lacking (15, 16).

The objective of the present study is to find out the prevalence of utilization of health care services as well as examine the different factors associated with utilization of health care services among elderly people in rural area setting. After finding out the different factors influencing the utilization of health care services, this study supports policymakers by providing real-life situational data. The utilization of health care services in this study refers to “visiting health care facilities to seek health care services one time or more than one time within one year” by elderly people.

Healthcare service utilization among the elderly is influenced by various factors, including demographic characteristics like age, gender, income, ethnicity, education, religion, marital status, employment status, annual income, decision-making, allowances, and family size (11, 15, 17, 18). While studies (11, 15, 17, 18) have examined healthcare utilization among the elderly in urban areas, there is limited evidence from rural settings. Therefore, this study aimed to investigate the factors influencing healthcare service utilization among the rural elderly population of Nepal.

## 2 Methods

### 2.1 Study design

A mixed method study, convergent parallel design, was used to identify and elaborate the influencing factors for health care services utilization among the elderly from October 2021 to March 2022. The convergent parallel design in mixed methods research in this study starting with a quantitative study to establish patterns and relationships, followed by a qualitative study to delve into deeper meanings and contexts. This approach combines the strengths of both quantitative data for structured understanding and qualitative insights for nuanced exploration. By integrating findings from both phases in parallel, this approach not only enhances the validity and reliability of the study but also enriches interpretations by integrating numerical data with qualitative insights. We used a standard cross-section design by strengthening the Reporting of observational studies in Epidemiology (STROBE) Checklist (Supplementary Appendix-S1) for the quantitative part and consolidated Criteria for reporting Qualitative research (COREQ) guidelines for the qualitative aspect of the study (Supplementary Appendix-S2).

### 2.2 Study population

The 2011 Nepalese census reported a population of 13,403 in the area, which falls under the jurisdiction of Bihadi Rural Municipality with a total area of 44.80 square kilometers in Parbat district, Gandaki province, Nepal (19, 20). The study place is situated 49.7 kilometers away from the district headquarters, which lies within the province with a high elderly population density (21). We interviewed people aged ≥60 years and living in the same areas. We also defined exclusion and inclusion criteria for this study. Exclusion criteria included those who refused to participate, those with hearing or speech impairment, individuals with severe illnesses, and those incapable of responding due to speech limitation or non-verbal communication. Moreover, the person who could not communicate effectively during the data collection was excluded. Furthermore, age, gender, living arrangement (alone, with a spouse, or with children), and existing medical conditions were considered for qualitative data collection.

### 2.3 Sample size

Using a single population percentage formula and the following presumptions, the sample size was calculated: The proportion of elderly persons utilizing health care services was 70% in Pokhara Lekhnath metropolitan city of Nepal (21), 5% error margin, and a 95% confidence level were used. Thus, the calculated sample size was given by *n* = Z^2^p*q/e^2^ Sample size (*n*) = (1.962*0.7*0.3)/(0.05)2 = 323.

After considering the 10% non-response rate, the final sample size was 355. Similarly, for the qualitative portion, and we interviewed 18 respondents until the data saturation was achieved.

### 2.4 Sampling and data collection procedure

**Figure 1:**
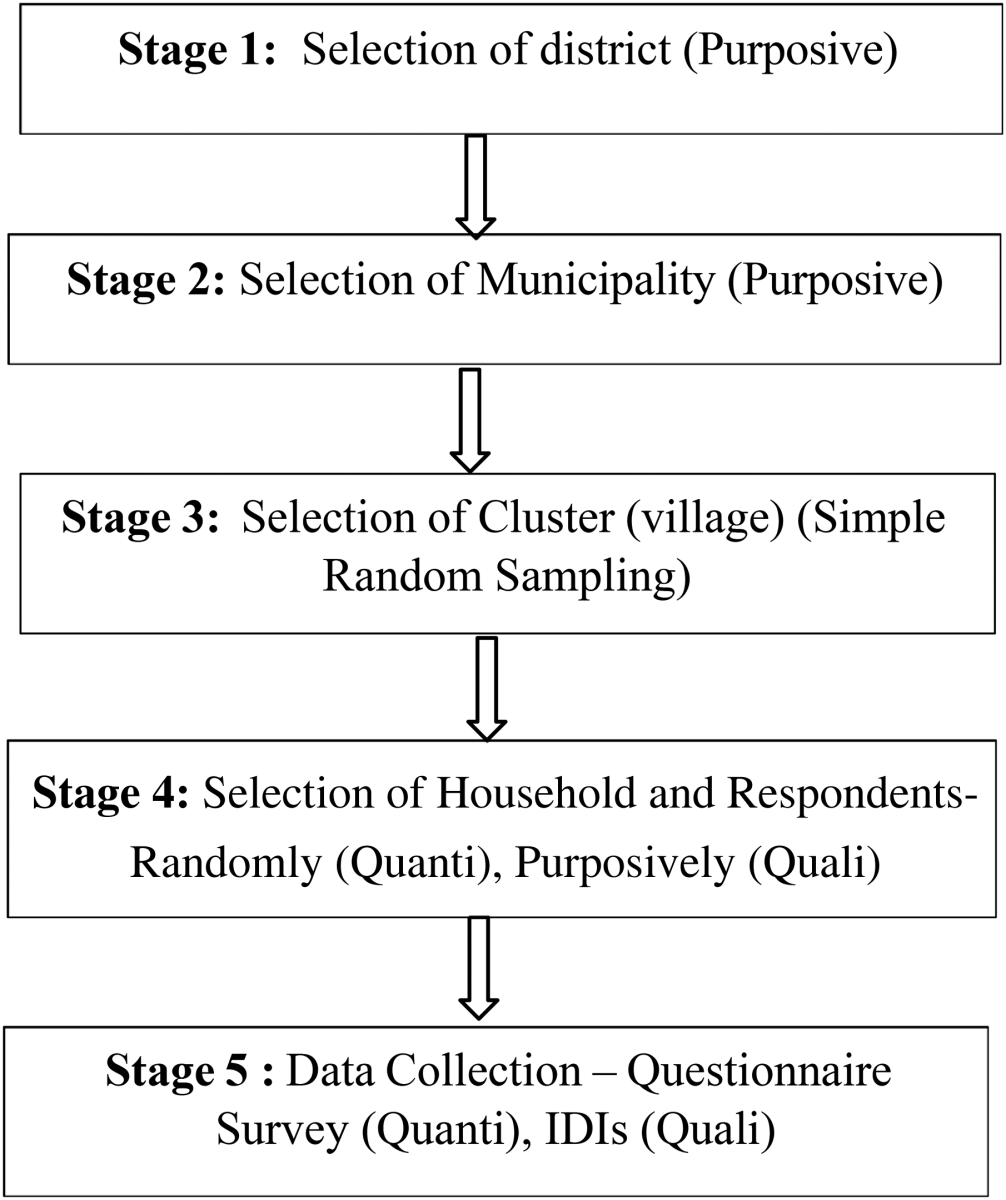
Flow diagram of multistage sampling technique.

The data collection tools were developed based on previous literature. Structured questionnaires for the quantitative studies and in-depth interview guidelines for the qualitative studies were used. The tools were pretested in the similar setting of the district. The questionnaire included socio-demographic factors such as age of the respondent, family type, education status, marital status, economic status, employment and ethnicity, healthcare service factors include nearest health care facility, reason for the health facility visit, satisfied of health care services, available of health status, able to afford health care services and utilization of health insurance, family support decision to take health care services in family, distance and means of transportation and health status. Meetings with every ward chief were conducted, and a social map was created. A multistage sampling technique was used to collect the information (Figure 1). A meeting was conducted at the rural municipality office with the health chief to discuss approval before data collection. It was assured that the rural municipality's health section would support data collection.

Stage 1: Purposively, the district was chosen. The district was chosen through the use of purposeful sampling because Parbat is a special location that is remote from excellent medical services and a backward area without access to a road. This sets this location apart from other areas and districts. Purposive sampling is employed to identify members of a certain group who have a distinctive attribute.

Stage 2: Selection of municipalities: There are two municipalities and five rural municipalities in Parbat District. A purposive sampling method was used to select the rural municipality because Bihadi is more backward based on available health facility services and access, and the distance to a higher-level health center is more than 2 h from the place. There is limited road access for the people while accessing health services to all people.

Stage 3: Selection of (small village) cluster: There were six wards in all Rural Municipality. There were nine small villages in one ward. 30% of all (clusters) of each ward were selected randomly.

Stage 4: In each cluster, the first residence was picked using a simple random sampling method, and the house screening method was used to select the elderly person for the whole study; in cases where a household had two or more elderly people, a lottery-based method was employed to select one elderly individual for inclusion in the study randomly. If the chosen initial household had no elderly people, the study team proceeded to the next household in line. All elderly individuals who were capable of responding and expressed their willingness to participate were included in the study. However, during the data collection phase, twenty elderly individuals were unavailable for participation. Ten declined to respond during the interview, while the remaining ten were undergoing medical treatment and taking medication, rendering them ineligible for inclusion in the final data collection process.

Stage 5: At the last we collect the data from 355 respondents for the quantitative study. For in- depth interviews, a semi-structured interview guide was employed to collect the qualitative data as per our study is mixed method study. For the qualitative data we made some criteria to select the respondent. Such as age of the respondent, gender, family type, and having property or not. We recorded the voice of all 18 respondents as field note taking was done of each interview after the inform consent of respondents. Qualitative data was completed after all the respondent's response appear same (Saturation of information). Qualitative data collection was completed with saturation of information. Altogether, 18 interviews were conducted. Field note taking and audio recording of each interview with permission from the respondents were done.

One of the principal investigators KP- (Male, BPH) collected the data from the field on January 2022. The face-to-face interview was conducted among the 355 respondents in study site. Based on the interview questionnaire, the socio-demographic information of respondents was collected. The data collection tool was first pre-tested among the 30 respondents in the adjoining village excluded from the final analysis. Modification based on pre-testing was done in the questionnaire. During the questionnaire's development, experts’ suggestions were taken and incorporated into the final questionnaire. All the interview was conducted in the Nepali language.

The IDIs were conducted to explore the personal experiences, feelings, and factors that limit the respondent's utilization of healthcare services. A total of 18 IDIs were performed by one interview KP (Male, BPH). All the respondents provided informed consent for interviewing. KP is a public health graduate from Shree Medical and Technical College Chitwan, affiliated with Purbanchal University, Nepal. All the data were recorded during the interview, transcribed, and translated into English. KT (Male, MPH, MPhil) and DM (Male, MA, MPhil) guided KP for thematic and content analysis. KT and DM are experienced public health professionals from Nepal with ample knowledge in mixed method study design. KP also took field notes during the field works.

All the questionnaires and interview guides used in this study are included in the Supplementary Appendix-S3 *(quantitative questionnaire- Nepali and English version)* and Supplementary Appendix-S4 *(In Depth Interview Guide- Nepali and English version)*, respectively. Both the questionnaire and guide were translated and validated before the data collection.

### 2.5 List of variables

#### 2.5.1 Dependent variables

Utilization of health services.

#### 2.5.2 Independent variables

Socio-demographic related characteristics- Age, Gender, Ethnicity, Family types, Education, Employment, decision making for health care, family support, support types.

Health care Service related characteristics- Nearest health facility, satisfaction with health services, availability of staffs, availability of medicine, attitude of health staffs, afford available health services, means of transportation.

### 2.6 Identified themes

We identified five themes, including the health status of the elderly, problems arising during utilization of health care services, support from family, health services, and the need to use health care services for elderly people. The details of the identified themes and subthemes are listed in Supplementary Appendix-S6.

### 2.7 Statistical analysis

#### 2.7.1 Quantitative analysis

Data was entered and analyzed in [SPSS] version 20 and R version 4.2.3. The frequency distribution of categorical variables was computed using descriptive statistics, and a chi-square test and then binary logistic was performed to test the relationship between variables. All the socio-demographic variables and health -service related variables were taken into logistic regression. Four different models for health service utilization were computed. The Akaike Information Criterion (AIC) score was computed and compared for different models. The model with low AIC score was mainly discussed in this paper.

#### 2.7.2 Wealth indexing

The wealth index was developed using Principal Component analysis (PCA) of several household things to measure the standard of living of a rural community's elderly. I.e., keeping of radio, TV, fan, watch, mobile and dwelling characteristics- material of roof, walls, and floor, type of toilet, and access to safe and clean drinking water and electricity. At last, the wealth index was categorized as a rich, medium, and poor group.

#### 2.7.3 Qualitative analysis

All recorded interviews were first transcribed in Nepali language. The transcription was then translated into the English language. The desk review of the information was done. We became familiar with verbatim during the desk review. After familiarizing the information, we need the data and themes to be prepared. Thus, thematic analysis was done in the qualitative part. Information supported through the quantitative analysis was mentioned.

### 2.8 Ethical statement

The Institutional Review Committee (IRC) of Shree Medical and Technical College approved (Ref # SMTC-IRC-20211009-01) this study. The administrative departments of the data collection sites provided the letter of permission for data collection. Informed written consent was taken from each respondent of the study. They were briefed about the information regarding the study's objectives, harm, and benefits of taking part in the survey. The data collection was entirely voluntary, and one could leave the study at any time without answering some or the whole questions. On the data collection form, there were no personal identification details. Except for the investigators, no one else had access to the collected material, which was kept private and anonymous.

## 3 Results

### 3.1 Quantitative information

Table 1 shows health care services utilization, and health status of elderly of 355 respondents. More than half (65%) of the respondents utilized health care services in the last 1 year including private, government clinic and other health care facilities, (39.7%) respondent had good health status, and (69.5%) respondent had chronic disease.

**Table 1:** Health services and status of respondents (*n* = 355).

Characteristics	Frequency	Percent
Utilization of healthcare services in the last year		
No	123	35
Yes	232	65
Health status of elderly		
Good	141	39.7
Ok	136	38.3
Bad	78	22.0
Type of disease (#190)		
Chronic	132	69.5
Acute	58	30.5

Among 355 respondents, a majority of 70--79 years old, the average age was 72.61 years (Table 2). Of them, about half (49%) identified as Brahmin/Chhetri and 50.7% of them were men and 51.8% were part of nuclear households, 68.2%, a sizable majority, had never attended school. 62.3 percent of people made most of their own decisions regarding their health care, 57.5% were happy with the assistance they received from family, and 34.4% specifically received support for their treatment from family members.

**Table 2:** Socio-demographic characteristics of respondents (*n* = 355).

Characteristics	Frequency	Percent
Age (year)*		
60–69	133	37.5
70–79	152	42.8
80 or more than 80	70	19.7
Gender		
Male	180	50.7
Female	175	49.3
Ethnicity		
Brahmin/Chhetri	167	47.0
Advantaged Janajaati	48	13.5
Disadvantaged Janajaati	72	20.3
Dalits	68	19.2
Type of Family		
Nuclear	184	51.8
Joint	171	48.2
Education		
Illiterate	242	68.2
Literate	113	31.8
Educational Status *n* = 113		
Basic level	84	74.3
Secondary	29	25.7
Employment Status		
Unemployed	321	90.4
Employed	34	9.6
Decision maker for health service in family		
Self	221	62.3
Spouse	41	11.5
Children and their spouse	93	26.2
Satisfaction from family support		
Satisfied	204	57.5
Neither satisfied nor dissatisfied	54	15.2
Dissatisfied	97	27.3
Type of support by family member (#256)		
Financial support	53	20.7
Looking after parents	35	13.7
Support in maintaining personal hygiene	38	14.8
Support in food	23	9.0
Support in Treatment	88	34.4
Help in farming	19	7.4
Distance to nearest health facility		
0–15 (minutes)	154	43.4
16–30 (minutes)	117	33
More than 30 (minutes)	84	23.7

* Mean age = 72.61, Min = 60, Max = 97.

Experiences that respondents had during the health service utilization is presented in Table 3. Of those who visited health posts, 50.7% said they were satisfied with the services, making up the majority (57.7%). Less than half (43.9%) of respondents said there was enough medicine available, whereas the majority (63.9%) thought medical personnel was always present. Of the respondents, 61.4% reported that the staff members were kind and 72.4% said they could afford the services. Despite these advantages, 90.4% of people walked to the medical institution and 92.7% did not use health insurance. These findings point up both the good and bad aspects of the provision of healthcare services.

**Table 3:** Health care services utilization among the respondents (*n* = 355).

Health services factor	Frequency	Percent
Nearest health facility		
Health post	205	57.7
Government hospital	33	9.3
Private clinic	117	33.0
Satisfied with available health care services		
Satisfied	180	50.7
Dissatisfied	175	49.3
Availability of health staff		
Always	227	63.9
Not always available	128	36.1
Availability of medicine		
Sufficient	156	43.9
Not sufficient	150	42.3
Not available	49	13.8
Attitude of health staff		
Good	218	61.4
Not good	137	38.6
Afford available health care service		
No	98	27.6
Yes	257	72.4
Utilization of health insurance		
No	329	92.7
Yes	26	7.3
Means of transportation		
On foot	321	90.4
Bus	34	9.6

We examined the relationship of individual factors, socio-demographic factors (Model I), health service-related factors (Model II), socio-demographic and health service factor together (Model III), factors from stepwise logistic regression (Model IV) (Table 4). In this table, only the significant factors in individual factors, model III and stepwise logistic regression model is presented, details table is available (Supplementary Appendix-S7). People of advanced Janajati (OR, 2.804, 95% CI 1.289–6.808), visiting private clinic (OR 1.864, 95% CI 1.133–3.123), availability of health staffs (OR 4.165, 95% CI 2.186–8.196), able to afford health care (OR 2.195, 95% CI 1.360–3.547), Bus as means of transportation (OR 2.676, 95% CI 1.148–7.324), who had bad health status (OR 2.227, 95% CI 1.431–3.491) had higher odds of health service utilization. Moreover, people dissatisfied from family support (OR 0.440, 95% CI 0.266–0.727), not sufficient medicine availability in Health facility (OR 0.400, 95% CI 0.234–0.674), not available any medicine (OR 0.365, 95% CI 0.138–0.085), not good attitude of health staffs OR 0.454, 95% CI 0.290–0.707), 16–30 min of distance to health facility (OR 0.453, CI 0.271–0.751) had lower odds for health service utilization. Among the model, model III is discussed based on low AIC score.

**Table 4:** Factors influencing health service utilization among the respondents (*n* = 355).

Characteristics	Crude odds ratio	Model I socio-demographic	Model II health service	Model III socio-demographic and health service	Model IV forward stepwise logistic regression
	cOR	aOR (95%CI)			
Ethnicity	Brahmin/Chhetri (Ref)				
Advantaged Janajati	**2.804** (**1.289–6.808)**	3.549 (1.273–11.855)		**3.728** (**1.062–15.887)**	
Disadvantaged Janajati	0.881 (0.50–1.567)	0.774 (0.346–1.753)		0.457 (0.165–1.254)	
Satisfaction from family support	Satisfied (Ref)				
Neither satisfied nor dissatisfied	0.732 (0.390–1.401)	0.955 (0.445–2.117)		1.212 (0.456–3.413)	
Dissatisfied	**0.440** (**0.266–0.727)**	1.241 (0.155–26.128)		1.301 (0.106–37.167)	
Nearest health facility	Health Post (Ref)				
Government hospital	0.653 (0.311–1.375)		0.111 (0.031–0.345)	**0.046** (**0.009–0.193)**	**0.294** (**0.125–0.686)**
Private clinic	**1.864** (**1.133–3.123)**		0.781 (0.41–1.483)	0.503 (0.202–1.241)	1.025 (0.575–1.837)
Satisfied with available health care services	Satisfied (Ref)				
Dissatisfied	**0.253** (**0.157–0.402)**		0.394 (0.216–0.713)	0.488 (0.214–1.091)	
Availability of health staffs	Always (Ref)				
Not always	**4.165** (**2.186–8.196)**		0.359 (0.192–0.663)	**0.375** (**0.147–0.931)**	
Availability of medicine	Sufficient (Ref)				
Not sufficient	**0.400** (**0.234–0.674)**		0.534 (0.268–1.052)	**0.372** (**0.143–0.924)**	**0.34** (**0.19–0.592)**
Not available	**0.365** (**0.138–0.085)**		0.055 (0.018–0.153)	**0.014** (**0.002–0.068)**	**0.026** (**0.009–0.066)**
Attitude of health staffs	Good (Ref)				
Not good	**0.454** (**0.290–0.707)**		1.599 (0.829–3.176)	**2.943** (**1.15–8.046)**	
Able to afford health care service	No (Ref)				
Yes	**2.195** (**1.360–3.547)**		1.573 (0.855–2.875)	1.94 (0.797–4.793)	
Means of transport	On foot (Ref)				
Bus	**2.676** (**1.148–7.324)**		3.455 (0.884–16.55)	**8.397** (**1.587–55.091)**	
Health Status	Good (Ref)				
Bad	**2.227** (**1.431–3.491)**		1.922 (1.106–3.364)	0.861 (0.375–1.924)	
Distance to health facility	0–15 min (Ref)				
16–30 min	**0.453** (**0.271–0.751)**		0.407 (0.209–0.776)	0.658 (0.256–1.669)	
>30 min	0.750 (0.423–1.341)		0.845 (0.373–1.948)	0.926 (0.299–2.983)	
*Akaike information criterion (AIC)	**–**	**325.26**	**353.36**	**273.1**	**383.84**

Model I- Socio-demographic, Model II- Health Service, Model III- Socio-demographic and Health Services, Model IV- Stepwise Logistic Regression for all factors.

Odds Ratio(OR) > 1 indicates increased odds for the outcome, while an OR < 1 indicates decreased odds. If the 95% CI does not include 1, results are considered as statistically significant.

If we consider all the factors influence on health services utilization (Model III), ethnicity (OR 3.728, 95% CI 1.062–15.887), not good health status (OR 2.943, 95% CI 1.15–8.046), bus as means of transportation (OR 8.397, 95% CI 1.587–55.091) had higher odds of health service utilization. Moreover, government hospital (OR 0.046, 95% CI 0.009–0.193), not always available health staffs (OR 0.375, 95% CI 0.147–0.931), not sufficient medicine (OR 0.372, 95% CI 0.143–0.924), not available medicine (OR 0.014, 95% CI 0.002–0.068) had lower odds for health service utilization.

At the last, based on statistical modelling (Model IV), we determined the major factors for health service utilization is health facility and availability of the medicine. The model showed that government hospital (OR 0.294, 95% CI 0.125–0.686), not sufficient medicine availability (OR 0.34, 95% CI 0.19–0.592), not available medicine (OR 0.026, 95%CI 0.009–0.066) had lower odds for health service utilization.

### 3.2 Qualitative information

We only interviewed 18 respondents in this study, which showed nearly similar results to quantitative data for health service utilization. The details of the socio-demographic information of the respondents have been listed in Supplementary Appendix-S6.

### 3.3 Summary of qualitative findings

The respondents reported their health status as falling somewhere between good and bad. A significant number of respondents reported having frail bodies and encountering more challenges when living in nuclear family settings. Difficulties in mobility, lack of transportation options, limited family support, extended waiting times at healthcare facilities, financial constraints, low awareness about health insurance, and individual attitudes, as well as attitudes toward healthcare services, emerged as factors influencing health service utilization.

Furthermore, financial independence appeared to play a crucial role in enabling respondents to access healthcare services. Additionally, community support and the availability of healthcare facilities within reasonable proximity were identified as important factors facilitating increased utilization of healthcare services among the respondents.

#### 3.1.1 Health Status of elderly

Most of the female respondents [15/18] said that their health status was neither good nor bad. Many respondents reported having experienced previous illnesses for which they had received medical treatment. However, despite taking medication, they continued to face health issues, including headaches, fever, excessive sweating, eye discomfort, leg and back pain, and difficulties with standing and breathing properly. Some respondents mentioned ongoing health conditions, such as pneumonia and asthma, for which they were currently undergoing treatment. On the other hand, a few male respondents expressed that their health was in good condition, and they were actively engaged in strenuous activities, managing tasks independently.

“My health is currently unstable with persistent pain in my eyes, legs, and back, along with pneumonia, asthma, headaches, fever, sweating, difficulty breathing, and the ongoing need to cook for myself despite inadequate relief from medication.”

– IDI06

“I live alone. I am fine. My health status is good. I cook food by myself. My son is going to a foreign for the job. That’s why I need to do all the work by myself, and I don’t have to take medicine until now. I can walk to work all day. My eyes are good. I eat and sleep in time.”

– IDI11

#### 3.1.2 Problem arises during the utilization of health care services

##### 3.1.2.1 Personal problem

All the respondents consistently mentioned that they experienced difficulties in walking to access healthcare facilities. They expressed that they struggled to stand properly when they were unwell and felt that their bodies were burdened by heaviness. Many also described having weak nerves, with their nerves appearing thin like hair on their skin. Financial constraints were a significant barrier for them, as they lacked the necessary funds to access healthcare services, purchase medications, and buy essential items like food and other necessities. In some cases, they resorted to taking loans in order to seek medical checkups and treatment at hospitals.

“I have personal problems. I am suffering from rheumatism, and I cannot walk properly. That’s why I cannot stand for a long time. I feel that my body is heavy. I live alone. Sometimes I have financial problems due to that I can’t go to the hospital for regular check-ups.”

– IDI17

##### 3.1.2.2 Family problem

The majority of the [16/18] respondents reported that they did not have family members along with them to support them in going to the hospital even if they did not have anyone to provide water when they felt sick. The respondents faced significant challenges in accessing hospital checkups and utilizing healthcare services due to financial constraints. Many of them revealed that their children were not providing support, particularly during times of illness, and a majority of their children were living apart from the family. This left them living alone and responsible for work and sustenance, which exacerbated their economic difficulties. Consequently, most elderly respondents were unable to afford timely medication and were reluctant to seek healthcare services, impacting their overall quality of life.

However, a few respondents shared a different experience, noting that they did not encounter family-related obstacles when visiting healthcare facilities. Their children supported them by providing financial assistance and sought loans from others to finance their medical treatments, ensuring better access to healthcare services.

“Although I have three sons living abroad, they neither contact us nor provide financial support for our medical needs and daily necessities, leaving us struggling without access to healthcare.”

– IDI06

“I have three sons of which two sons were died. Beside them, one son is alive but he is living separately with his family. When we are sick, they don’t come to visit for us. We don’t have a family member to give water at the time of sick and we live alone. We don’t have money that’s why no one supports us to go to hospital for checkup due to that I don’t go to hospital to utilize health care services.”

– IDI03

“My family supports me for checkup. They encourage me to go to hospital. They give money for treatment and to buy food which I want. All my sons and daughters-in-law are good. They go with me to the hospital for my checkup.”

– IDI16

##### 3.1.2.3 Community problem

All respondents indicated no road leading to the healthcare facilities, and they faced challenges walking on rugged trails. No consistent transportation options resulted in longer travel times to reach healthcare facilities. Tragically, due to the lack of accessible roads, some individuals lost their lives while transported to the hospital in bamboo baskets. Additionally, they mentioned that their neighbors did not provide assistance when they fell ill, and mutual support was lacking in challenging circumstances.

“Here is not a road to go to a health facility. We need to use the trail, which is more dangerous. We need to walk through the cliff while going to health facilities. During the emergency, some serious patients died on the road due to the unavailability of transportation. We must take the patient to the hospital by carrying a bamboo basket. Our neighbor doesn’t help us. They don’t care about each other. They never lend us money at the time of the most difficult situations. Sometimes, we need to call the doctor at home, and for that we need to pay more fees. Due to the insufficient money, we cannot utilize the health care services.”

– IDI15

##### 3.1.2.4 Health facility problem

Half of the respondents [9/18] reported that medicine was insufficient for us, and some drugs were not working effectively. According to their perspective, they believed their lack of literacy resulted in the healthcare staff providing them with low quality medications. They expressed concerns about the inadequate management of medicines and the absence of qualified healthcare professionals with MBBS degrees, which necessitated traveling to a distant clinic. They found that the clinic's medication was effective. Unfortunately, there was no x-ray machine for internal examinations, and laboratory facilities for blood, sputum, stool, and urine tests were unavailable. However, it's worth noting that half of the respondents appreciated the management of the healthcare facility, the availability of medicines, and the provision of minor surgical procedures there.

“I think as we are illiterate elderly people, that’s why damaged and expired medicines were given to us in the health post. That’s why I don’t want to go for checkups. Here, the management of health facilities is not done properly. Due to some financial problems, I must go to a health post. Good doctors are not available for checkups. There is no availability x-ray machines and other kinds of laboratory facilities.”

– IDI13

“There is no problem in a health facility. I used to work there. We used to manage everything, such as management of medicine and staff. All medicines were available in the clinic.”

– IDI17

#### 3.1.3 Support from family

More than half [12/18] of the respondents from nuclear families reported that they did not get support from the family as their children started to live separately. In contrast, few of the respondents from atomic families and joint families said that they got support from their offspring. The respondents mentioned that their sons and daughters-in-law played a crucial role in caring for them. Their children provided financial support and accompanied them to the hospital, assisting in their treatment and helping with agricultural work in the fields.

“I have children, but they live separately after the quarrel and have their own lives. They don’t talk to us. They never help us, even when we are sick. They didn’t ask us about our health, if we were in good condition or bad condition. I don’t hope that they will help us in future. We will work and earn money until we can and save money to go to the hospital to check my health.”

– IDI03

“My son and daughter-in-law are living out of the district for jobs. They helped me when I was sick. They took me to the hospital for a checkup and to treat my disease. They talked to my doctor, supported me financially, and helped me with medication. My daughter-in-law helped me to maintain personal hygiene. My other son stays with us at home. He helps in the field and lives at home when we went outside for checkups.”

– IDI01

#### 3.1.4 Health services

##### 3.1.4.1 Waiting time to access health care services

The majority of the respondents (16/18) expressed that sometimes they had to wait a long time to access health care services, and sometimes they got immediate access to health care services as one person reported that the long waiting time to access health care services made patients frustrated and discouraged them to utilize health care services. However, there is a provision of no waiting time while utilizing health care services in Nepal.

“I don’t need to wait till a long time. Doctors give priority to elderly people, but sometimes there is a long queue because of that, I need to wait a long time to access health care services.”

– IDI05

“That was a big hospital in which many people visited for health facilities. Due to the long queue, I got frustrated and discouraged to utilize the available health care services.”

– IDI01

##### 3.1.4.2 Time to checkup

The majority of the respondents (15/18) reported that the doctors provided excellent treatment and prescribed medications as needed. The effectiveness of the prescribed medications varied, with some medicines producing rapid results while others did not. Respondents mentioned that doctors typically thoroughly examined and prescribed medication within approximately 5–10 min.

“They treat so well. The doctor always checks my pressure. After that he prescribes medicine for me. He also suggests how to take medicine and which food I should eat. Sometimes medicine works, and I get well, but sometimes medicine doesn’t work. For all processes, it takes around 5–10 min.”

– IDI09

##### 3.1.4.3 Afford to get health care services

The majority of the respondents (14/18) reported that they could not afford available healthcare services due to lack of money. Respondents mentioned that they engaged in fieldwork to alleviate financial difficulties. Occasionally, they had to take out loans to access healthcare services at health facilities. Some respondents stated that they could afford healthcare services through their pensions and allowances, while at other times, their children provided them with financial assistance for personal expenses.

“I have a difficult situation due to lack of money. I do labor work that is why I have low income of source to checkup sometimes. We need a lot of money that we can’t afford, so we need to ask for money from others. If someone lends us money, we go for checkups at the health facility.”

– IDI07

“My sons give me money to buy my desired things, and my husband has a pension and I have an allowance. I can afford health care services.”

– IDI14

##### 3.1.4.4 Utilization of health insurance

Most respondents (16/18) reported that they did not utilize health insurance because they were unaware of the health insurance scheme and facilities. They said they had never received any recommendations or guidance on utilizing health insurance, so most of them hadn't used this service. However, one person shared a positive experience, expressing satisfaction with health insurance. This individual had accessed free healthcare services at a large hospital through their health insurance coverage.

“I don’t utilize health insurance. I don’t know about free health care services and about these insurances. No one has suggested me about health insurance.”

– IDI02

“My son works in a health post. He knew about these schemes; he made health insurance services for all family members, even if they lived outside the home. I got many free health care services, including x-rays and different tests such as blood and urine. I am so happy and satisfied to use these services.”

– IDI17

##### 3.1.4.5 Attitudes of health staff towards elderly people

Majority of the respondents (14/18) expressed that they felt good to talk to doctors. The healthcare providers were commendable in their approach, displaying a positive attitude towards elderly individuals. They were able to explain medication usage and provided comprehensive information about the factors contributing to their health situation. They communicated politely and with gentle voices, maintaining a respectful demeanor even when they were doctors themselves.

“They have a good attitude towards elderly people like me. Doctors talked with me so politely, and he had a great capacity to clarify everything such as medicine, how to take it? Whether food should be eaten or not, they suggest everything. That’s why I prefer to go to the clinic to use health care services.”

– IDI17

##### 3.1.4.6 Elderly perception of quality healthcare services

A few respondents (7/18) expressed mixed feelings about their healthcare experiences, as they noted that medication was effective at times but not consistently so. However, they acknowledged that health staff members excelled in treating minor wounds and warts. Additionally, they reported that most people were content with the services provided by the healthcare facilities, which encouraged many elderly individuals to visit these facilities regularly.

“I am satisfied with this clinic’s health care service. Sometimes medicine work immediately, and sometimes doesn’t work. I don’t know why this happens. When my son had a big wound, he went there; they treated him so well. He and most of the people wanted to go there for checkups. The quality of health care services in that clinic is good.”

– IDI14

#### 3.1.5 Requiring needs to use health care services for elderly people

##### 3.1.5.1 Individual factor

Most respondents [15/18] reported that if they could walk and were strong enough, they would go to health for treatment. They expressed their desire for financial freedom, mentioning that if they had sufficient funds, they would spend them as needed, whether purchasing medicine or going for medical check-ups whenever they wished. They also stated that if they possessed the physical strength of a young person, they would prioritize saving money for future healthcare needs. Furthermore, they emphasized that if their hearing and mobility remained intact, they could visit the hospital regularly for routine check-ups.

“If I were not suffering from this disease, I would go to the health facilities quickly. I would use health care services frequently. If I had money, I could go to the clinic soon. All elderly people would have access to a health facility if they had money. If they were strong enough, they would do work like when they were young.”

– IDI07

##### 3.1.4.2 Family factor

Respondents from nuclear families (only husband and wife or single) reported that they would use many health care services if they lived together with family. Children would help us to go to a health institution for treatment if we lived with them. It would have been easy for everyone. If their children paid for treatment and looked after the housework, they could go for treatment whenever they wanted. Support from family members, close acquaintances, and neighbors by providing information, helping to reach health facilities and access health services was identified as a facilitator for health care service utilization. In contrast, neglect, and lack of love from family and friends were identified as barriers to health service utilization.

“I would use many health care services if I lived with my family. I would have used more healthcare services if the family managed the money. If our children cared for us and ensured the food was given to us when we were sick, hygienic people like me would often use health services.”

– IDI17

“Support was from my son; no one else provided any support. My son helps me and gives me everything.”

– IDI15

##### 3.1.4.3 Community factor

Most respondents [15/18] reported that the lack of road accessibility at specific locations has resulted in the tragic loss of life, and constructing roads in these areas could have prevented such incidents, even without access to public transportation. If the roads were easily accessible and the community had supportive neighbors, it would encourage people to utilize healthcare services more frequently.

“If there were a good road in the village for every elderly house, it would have been easy to go to health facilities to get health care services. The health care services would be a great use if the neighbors were helpful and dared to say that we would look after you go for treatment.”

– IDI02

##### 3.1.4.4 Health facility

Most respondents, specifically [17/18], reported that if the government had established hospitals, medical colleges, and improved road infrastructure in their area, it would have significantly facilitated healthcare access for both them and future generations. They also emphasized the importance of having well-educated doctors available locally. The availability of comprehensive healthcare services, including medication and diagnostic tools like x-rays, within their vicinity would have eliminated the need for extensive travel to access healthcare.

In some instances, the absence of healthcare workers significantly discouraged elderly individuals from seeking medical care. Additionally, the poor infrastructure, lack of specialized geriatric services and necessary equipment, and absence of emergency transportation facilities like ambulances deterred them from utilizing healthcare services.

“If the government had established a hospital, medical college, and road here, with adequate doctors, nurses, medicines, and an x-ray machine, accessing healthcare would be much easier and more frequent.”

– IDI02

"Due to the lack of basic infrastructure, health facilities, and geriatric specialists at our rural health post, we had to endure lockdowns, financial strain, and transportation issues to seek care at a larger hospital during the COVID-19 pandemic.”

– IDI01

## 4 Discussions

In this studies, we explored different factors which influence health services utilization among elderly. We tried to explore in several different ways, first we explored individual factors, second is socio-demographic factors, third socio-demographic and health service factors, the fourth model explored among all these factors which one is the best. Thus, based on the most influencing variables, we explored elderly are lesser likely to visit government health facilities, no availability of the medicine is the major factor. Moreover, if we dealt together with all variables at once, ethnicity, not having good health status of elderly, bus as means of transportation positively influence health service utilization whereas government facilities, availability of medicine adversely influence it. Thus, different modeling strategies in this paper clearly illustrates that there is influence of various factors whose intensity matters based on the socio-demographic or health service availability context. Therefore, it is more important to know the social context and we explored other factors through qualitative interviews. Long waiting time, availability of medicine, attitude of health workers, family support, distances are other determinants of health service utilization from qualitative interview.

The current study found that 65 percent of the respondents reported utilization of health care services. In contrast, another study in Pokhara-Lekhnath Metropolitan city of Nepal showed it was 70% (21). Over half, 57.7% of respondents visited the health post in the last year. Due to the free services of Health Post, the rate of utilizing Health Post services was higher in Ghana (22). There was a significant association between the types of health facilities and the utilization of health services in our study. Similarly, when we compared the literature and this study, we find the contrast between them (22). Similar results were found in qualitative as the results showed the utilization of health care services, where health service utilization was 68% in Dhulikhel Municipality, Nepal (18, 23). Both studies showed that the utilization of health care services is still low, so the government should focus on elderly-friendly health services.

As, we explored in this study, ethnicity is the factor for health service utilization from model III and individual regressions. These findings were similar to the previous studies conducted in Nepal, meaning rural lower caste people still do not utilize health care services (15). These factors were further validated with our qualitative findings. Where age, marital status, sex, and education of the elderly showed that there is no association (11, 15, 18). There are also some contradictions regarding finding of gender (24). The discrepancy in these findings may be due to the differences in the geographical distributions, costs, travel distance, and policies of the different places according to local level. The major factors for such discrepancy is how you analyzed these factors, therefore understanding of the context is very important.

Here, we did not report any association between wealth and health service, the reason can be free availability of health care services in health post. A previous study done in rural places in the Chitwan district also showed the same result (25). Still, a study was conducted in Pakistan (26), and another study completed in Ghana showed a different effect (27). This mixed result means that in other countries, due to different rules and regulations, wealth status may or may not affect healthcare services’ utilization. Family support was considered an essential factor for health service utilization. We reported no significant difference is reported in family types contributing to health service utilization statistically however from qualitative aspect it is still important to have family support for elderly for better health care and health aging. Respondents said that their children supported them by giving them money, and they helped respondents by going to the hospital with them and supported them in treatment and assisting in the field. This finding of our study reported similarities with the Asante Akyem North District- Ghana study (28).

Our findings shows that there are higher number of elderly people who are belongs to underprivileged group (Advantage Janajati, Disadvantage Janajati and Dalit). Similar study was done in western part of Nepal which results are similar with our studies (16, 29). This may be due the presence of higher number under privileged group and people from lower socioeconomic backgrounds may face barriers to accessing quality care due to financial constraints or lack of insurance (30) The observations show that the nearest health facilities had less likely to utilize health care services than health post. The number of elderlies who utilized health care services from health post was highest compared to government hospital and other. This may be due to the distance from the home of elderly. Similar, findings are found out in previous study which was done in Manag rural; part of Nepal (15). This is probably because Nepal is geographically diverse place due to that still there is no road to reach to health facility and people prefer to walk by their foot to access health care services which is evident by our qualitative findings. Health post is near, cheap and they feel secure and confident to share their health issues with health staff. Regarding satisfaction with available health care services our observations show that elderly people who are dissatisfied with available health care services were less likely to utilize health care services compared to those who were satisfied with the available health care services. Satisfaction with health service utilization was very important which further built trust for further use. Aging people who were happy with health care services were two times more likely than those who were dissatisfied with public health care services. There was an association between satisfaction with available health care services and utilization of health care services, whereas the study conducted in Butwal was (*p* > 0.05), which means the findings contrasted (14). The reason for the contrast between our research and the Butwal study might be rural and urban areas. In urban areas, there are many health facilities available, so it doesn't affect the utilization of health care services in urban areas like Butwal. It is the same as the finding observed in our study contrasted with that found in Manang's study (*p* = 0.211) (4, 31) Similarly, the study conducted in Dhulikhel municipality found that there was no significant between satisfaction of medical care services and utilization of health care services (*p* = 0.263) (18). Our study shows that elderly had less likely to seek health care services when there is unavailable of medicine. Previous finding which was done in Butwal shows that must of the elderly utilize health care services when the medicine are available which is contrast with our study finding.

Though, we did not find any relationship between wealth and health care in rural setting. A study from Dhulikhel municipality showed a contrast result, which strongly proves that in developing countries like Nepal, people use health care services after they feel sick (18). Wealth index is a major socio-demographic factor that determine the acceptability and utilization of available health care services. Our study result showed that those elderly who had poor wealth they were less likely to utilize health care services compared to those who had rich wealth. Previous study which was done in Pakistan also showed the similar results as like our study (26, 32). Previous qualitative study which was done in nigeria shows the similar result that people have low fund that's why they don't seek the health care services (33). This clearly indicate the true picture of inequality in utilization of health care services by poor wealth and rich wealth elderly in Nepal. In our study showed the high number elderly people who visited the health post to utilize health care services.

We reported access to health facility is the factor either by qualitative or quantitative results. This finding contradicted other studies in Manang and other developing country, Ambo Town, West Shoa Zone, Oromia, Ethiopia (15, 24, 32, 34). Parbat being in a hilly region, there are more geographical obstacles and inaccessible roads to reach health facilities. Elderly is the most fragile state in which older people can't walk during their more senior stage (24, 34). Due to that, they were less likely to utilize health care services, but in the case of Ethiopia, that study was done in a town, that's why there is more access to transportation so people can easily reach the hospital when they want. All respondents reported that they were too frail to walk to the health facilities. This is associated with the utilization of health care services. Similar findings were found compared to the study conducted in Korle-Bu Hospital, Accera (35). All respondents reported that there was no road to reach the health facility. They had difficulty to walk on trails. Previous study which was done in Nigeria showed that elderly people who didn't have transportation facilities had less likely to utilize health care services (36). Due to inaccessible roads and geographical areas, some people died on the road and were taken to hospital by carrying a bamboo basket as reported by qualitative findings. In contrast, the study conducted in Iran showed Similar Findings (37). Older adults were utilizing more health care services where health staff were always available compared to those where health staff were not available. A study conducted in Ethiopia also supports these results (32, 38, 39). Medicine is also essential during the treatment. Without medication, health workers could treat elderly people. If there could be available all prescriptions, the elderly would more utilize health services. The quality of health service providers, such as communication skills, availability of sufficient medicine, time to take health care services, time for checkups, satisfaction with available health services, and availability of health services were associated with the utilization of health care services. A qualitative study conducted in Nepal found that the quality of healthcare services was closely related to the utilization of healthcare services (40). This finding was corroborated by a qualitative case study in Lisbon, Portugal (41). The result of this study is slightly similar to the survey which was conducted in Ghana (42, 43). This Study's findings were identical to the previous study's (long waiting to access health care services, lack of medicine, and poor attitude of Health staff) (44). About four among ten had self-reported good health status. Through the analysis of qualitative data, we found that elderly people when their health status was bad they tend to utilize more health care services. In this way, the study, which was conducted in Northern Taiwan, found that elderly people whose health status was good were less likely to utilize care services than those with bad/not good health status. This study can also support that people dislike using available healthcare services when their health is excellent (45). A previous study conducted in Uberaba/Minas Gerais showed an association between the use of healthcare services and self-reported morbidities. Likewise, this study found that the presence of different types of disease and utilization of health care services was significantly associated (34). A study conducted in Brazil found a close association between health status and utilization of health care services. This may be because as more people live longer, chronic diseases, the most common conditions of middle and old age, have emerged as significant causes of disability and functional dependency requiring services (35). The results showed that most respondents’ health statuses were neither good nor bad. This study explored the health status of the elderly, and the health status of the elderly people is associated with the utilization of health care services. Similar findings were found compared to the study conducted in Korle-Bu Hospital, Accera (36).

Furthermore, in this study, we did not focus on diseases such as diabetes, hypertension, etc. Still, we relied only on self-reported health condition where the respondents rated their health status as neither good nor bad. Therefore, we can't comment on individual health status of elderly. As recommended by other studies, the quality of life of the elderly can be another essential aspect that needs to be further discussed in terms of physical, psychological, and environmental health (46). Thus, it is important to examine the relationship between quality of life and health service utilization.

## 5 Strength and limitations

The major strength of our study is we relied on mixed methods. Though, there are several factors influencing health care utilization. We reported health medicine and nearest health facility is the major factors which is further evident by qualitative findings. Next, we tried to explore the influence of individual factors and how collectively they influence it, thus understanding of the context is very important.

This study was done in a rural Parbat district municipality with few health facilities and a high elderly population. At the same time, this might restrict the findings’ applicability to other settings in the Parbat district. Respondents self-reported their health status and use of healthcare, which may not accurately represent the parameter. Self-reported health information has, however, become a widely used strategy in practice, mainly when few resources are available. There could occasionally be recall bias, particularly for wealth status. The most sophisticated method for determining the precise level of respondents was used in this study: wealth indexing.

The relationship between aging and healthcare use in the context of Nepal has received attention however it is not sufficient. Our findings could serve as a foundation for future research in Nepal. Future research should estimate how many people use healthcare services based on their socio-demographic characteristics and location. Our studies will significantly assist future mixed-method research in social sciences and aging research.

## 6 Conclusions

In conclusion, this mixed-method study provided helpful insight into the socio-demographic and health service factors. The exact impact of each factors is influenced by context. Among all the factors, availability of medicine and nearest health facility is the most powerful factors. The trust between elderly and health facility is important to strengthen. The social context played a vital role in acknowledging the health care facility influence on overall satisfaction among elderly. Geriatric care training should be provided to health care providers with sufficient technology and skills to handle the health care needs of elderly. As this study only focused on a rural municipality of Parbhat district, further research is recommended exploring the factors, socio-demographic and all contextual other influencing factors. Large sample size, use of sophisticated technology, data collection covering multi geopolitical locations covering rural urban setting etc are recommended in further research.

## Data Availability

The original contributions presented in the study are included in the article/Supplementary Materials, further inquiries can be directed to the corresponding author.
