# Supplementary material for: Health care service utilization among elderly in rural setting of Gandaki province, Nepal: a mixed method study
Source: Front Health Serv. 2024 Sep 25;4:1321020. doi: 10.3389/frhs.2024.1321020 (PMC11462626; doi:10.3389/frhs.2024.1321020)
Supplement: Supplementary file 3 [file Table3.docx]

### Appendix. 3 Informed Consent And Questionnaire (English Version)

**PURBANCHAL UNIVERSITY**

**SHREE MEDICAL AND TECHNICAL COLLEGE**

**BHARATPUR CHITWAN**

Inform consent

Namaste,

I am kamal poudel. I am studying bachelor in public health in Shree Medical and Technical College Bharatpur Chitwan. At the time of study I am doing research in the topic of “**PREVALENCE AND FACTOR ASSOCIATED WITH HEALTH CARE SERVICES UTILIZATION AMONG ELDERLY”** I will keep secret your information and it is used for only study purpose and it is important for us. If you don’t want to participate in this interview you can quit during the interview. I hope your positive response.

1. Yes
2. No

**Name of interviewer:- ______** Date:-_____________

**Name of house owner:-_________________**

**Address:-_____________________**

**Phone no.:-_____________________**

**Signature of interviewer:-_________________**

**Signature of witness:-_____________________**

| **Q.NO** | | **Questions** | **Response** | **Skip** |
| --- | --- | --- | --- | --- |
| **1.      Social demographic factors** | | | | |
| 1.1) | Age | | …… Years |  |
| 1.2) | sex | | 1)Male |  |
|  |  |  | 2) Female |  |
| 1.3) | What is your marital status? | | 1)Married |  |
|  |  |  | 2) Unmarried |  |
|  |  |  | 3) widowed |  |
|  |  |  | 4) Divorced |  |
| 1.4) | What is your religion? | | 1) hindusam |  |
|  |  |  | 2) Buddhism |  |
|  |  |  | 3) Muslim |  |
|  |  |  | 4) Christian |  |
| 1.5) | How many family member do you have? | | ………….. |  |
| 1.6) | How many son and daughter do you have? | | 1) Son |  |
|  |  |  | 2) Daughter |  |
| 1.7) | What is your ethnicity? | | 1) Brahmin/Chhetri |  |
|  |  |  | 2) Advantages Janajaati |  |
|  |  |  | 3) Disadvantages Janajaati |  |
|  |  |  | 4) Aadhibaasi |  |
|  |  |  | 5) Dalit |  |
| 1.8) | Family type | | 1) Nuclear |  |
|  |  |  | 2) Joint |  |
|  |  |  | 3)Extended |  |
| 1.9) | What is education Status? | | 1)Illiterate |  |
|  |  |  | 2) ………… |  |
| 1.10) | What was your occupation? | | 1) Housewife |  |
|  |  |  | 2) Agriculture |  |
|  |  |  | 3) Services |  |
|  |  |  | 4) Labour |  |
|  |  |  | 5) Business |  |
|  |  |  | 6) Foreign Employment |  |
|  |  |  | 7) Unemployment |  |
|  |  |  | 8) Others |  |
| 1.11) | What is your employment status? | | 1) Unemployment |  |
|  |  |  | 2) Retayered |  |
|  |  |  | 3) Employment |  |
| 1.12) | | What is your main income source of your family? | 1)Housewife |  |
|  |  |  | 2) Agriculture |  |
|  |  |  | 3) Service |  |
|  |  |  | 4) Labor |  |
|  |  |  | 5) Business |  |
|  |  |  | 6) Foreign Employment |  |
|  |  |  | 7) Unemployment |  |
|  |  |  | 8) others |  |
| 1.13) | | How many month your main income sufficient to your family? | 1) Less than 6 months |  |
|  |  |  | 2) 6-12 months |  |
|  |  |  | 12) More than 12 months |  |
| 1.14) | | How much is your annual income? | …………. Nrs. |  |
| 1.15) | | Who afford your expenditure? | 1) Own self |  |
|  |  |  | 2) Family |  |
|  |  |  | 3) Relatives |  |
|  |  |  | 4) Friends |  |
|  |  |  | 5) others |  |
| 1.16) | | Do you have smoking habit? | 1) frequently |  |
|  |  |  | 2) sometimes |  |
|  |  |  | 3) Never |  |
| 1.17) | | Which smokes you do? | 1) cigarette |  |
|  |  |  | 2) Tambakhu |  |
|  |  |  | 3) Surti |  |
|  |  |  | 4) Paan |  |
|  |  |  | 5) others |  |
| 1.18) | | Do you have alcohol taking habit? | 1) frequently |  |
|  |  |  | 2) sometimes |  |
|  |  |  | 3) Never |  |

| **Wealth Index** | | |  | | |  |
| --- | --- | --- | --- | --- | --- | --- |
| 2.1) | What is your main sour of drinking water? | | 1) Own house water pipe | | |  |
|  |  | | 2) Neighbor’s house water pipe | | |  |
|  |  | | 3) Tube well | | |  |
|  |  | | 4) well | | |  |
|  |  | | 5) Public Tap | | |  |
|  |  | | 6) Spring Water | | |  |
| 2.2) | What type of toilet you use? | | 1) Modern Toilet | | |  |
|  |  | | 2) Borehole Toilet | | |  |
| 2.3) | What type of fuel you use to cook food? | | 1) electric | | |  |
|  |  | | 2) Gas | | |  |
|  |  | | 3) Biogas | | |  |
|  |  | | 4) Kerosene | | |  |
|  |  | | 5) Coal/Firewood | | |  |
|  |  | | 6) Animal Dung | | |  |
|  |  | | 7) Others | | |  |
| 2.4) | What your house's floor made of? | | 1) Mud/Sand/Dung | | |  |
|  |  | | 2) Bamboo/Straw | | |  |
|  |  | | 3) Wood | | |  |
|  |  | | 4) Tile | | |  |
|  |  | | 5) Cement | | |  |
|  |  | | 6) Others | | |  |
| 2.5) | What your house's roof made of? | | 1) Bamboo | | |  |
|  |  | | 2) Wood | | |  |
|  |  | | 3) Straw | | |  |
|  |  | | 4) Tin | | |  |
|  |  | | 5) Tile | | |  |
|  |  | | 6) Cement | | |  |
|  |  | | 7) Others | | |  |
| 2.6) | What your house's wall made of? | | 1) Mud/Wood | | |  |
|  |  | | 2) Bamboo/Mud | | |  |
|  |  | | 3) Stone/Mud | | |  |
|  |  | | 4) Ply | | |  |
|  |  | | 5) Cement Block | | |  |
|  |  | | 6) Brick | | |  |
|  |  | | 7) Others | | |  |
| 2.7) | Is there any facilities available there? | | 1)Facility of electricity | | |  |
|  |  | | 2)Facility of radio | | |  |
|  |  | | 3) TV | | |  |
|  |  | | 4) Mobile phone | | |  |
|  |  | | 5) other phone | | |  |
|  |  | | 6) fridge | | |  |
|  |  | | 7) Computer | | |  |
|  |  | | 8) watch | | |  |
|  |  | | 9) Others | | |  |
| 2.8) | Do you have private vehicle in your house? | | 0) No | | |  |
|  |  | | 1)Yes | | |  |
| 2.9) | If yes, which vehicle available? | | 1) Cycle/Riksaa | | |  |
|  |  | | 2) Motorbike/Scooter | | |  |
|  |  | | 3) Tempo | | |  |
|  |  | | 4) Car or Truck | | |  |
|  |  | | 5) Others | | |  |
| 2.10) | Do your family member names have field? | | 0) No | | |  |
|  |  | | 1)Yes | | |  |
| **Utilization of Health care services** | | | | | | |
| 3.1) | | Did you utilize health care services in last 1 year? | | 1) Yes |  | |
|  |  |  |  | 2) No |  |  |
| **Health Care Services** | | | | |  | |
| 4.1) | | Where do you go when you sick at first? | | 1) Traditional Healer |  | |
|  |  |  |  | 2) Health Post |  |  |
|  |  |  |  | 3) Primary Health Care Center |  |  |
|  |  |  |  | 4) Government Hospital |  |  |
|  |  |  |  | 5) Private Hospital |  |  |
|  |  |  |  | 6) others |  |  |

| 4.2) | How many time did you use health facility in past 1 year? | 1)….. Times |  |
| --- | --- | --- | --- |
| 4.3) | Why did you go to Health facility? | 1) Regular Checkup |  |
|  |  | 2) Health Problem |  |
| 4.4) | Are you satisfied by health care services of Health facility? | 1) Satisfied |  |
|  |  | 2) Neither satisfied nor dissatisfied |  |
|  |  | 3) Dissatisfied |  |
| 4.5) | How many time do you wait to take health care services of health facility? | 1) Long time |  |
|  |  | 2) Neither long nor immediately |  |
|  |  | 3) Immediately |  |
| 4.6) | Is there health staff available in your health facility? | 1) Always |  |
|  |  | 2) Sometimes |  |
|  |  | 3) Never |  |
| 4.7) | Is there medicine sufficiently available? | 1) sufficient |  |
|  |  | 2) Not sufficient |  |
|  |  | 3) Not available |  |
| 4.8) | How was your health staff attitude toward you? | 1) Good |  |
|  |  | 2) Ok |  |
|  |  | 3) Bad |  |
| 4.9) | Do they speak politely while giving health care services? | 1) Yes |  |
|  |  | 2) No |  |
| 4.10) | How is your Health staff communication skill? | 1) Good |  |
|  |  | 2) Ok |  |
|  |  | 3) Bad |  |
| 4.11) | How much time health take to check up? | ……. Minutes |  |
| 4.12) | Do you afford there available health care services? | 1) Yes |  |
|  |  | 2) No |  |
| 4.13) | What is your income source of your health expenditure? | 1) Insurance |  |
|  |  | 2) Allowance |  |
|  |  | 3) From children |  |
|  |  | 4) Pension |  |
|  |  | 5) others |  |
| 4.14) | Do you utilize health insurance? | 1) Yes | if No go to 5.1 |
|  |  | 2) No |  |
| 4.15) | What facilities do you get from health insurance? | ……… |  |
| 4.16) | Are you satisfied by health insurance's facility? | 1) Satisfied |  |
|  |  | 2) Neither satisfied nor dissatisfied |  |
|  |  | 3) Dissatisfied |  |
| 4.17) | Do you accept all health care services of health facility? | 1) Yes |  |
|  |  | 2) No |  |
| 4.18) | With you go to Health facility? | 1) own self |  |
|  |  | 2)spouse |  |
|  |  | 3) Son/Daughter in law |  |
|  |  | 4) Daughter/Son in law |  |
|  |  | 5) others |  |

| **Support from Family members** | | | | | | | |
| --- | --- | --- | --- | --- | --- | --- | --- |
| 5.1) | Who decide to take health care services in your family? | | | | | 1) own self | |
|  |  |  |  |  |  | 2)spouse | |
|  |  |  |  |  |  | 3) Son/Daughter in law | |
|  |  |  |  |  |  | 4) Daughter/Son in law | |
|  |  |  |  |  |  | 5) others | |
| 5.2) | Do your family support you at the time of taking health care services? | | | | | 1) Yes | |
|  |  |  |  |  |  | 2) No | |
| 5.3) | Are you satisfied by family support? | | | | | 1) Satisfied | |
|  |  |  |  |  |  | 2) Neither satisfied nor dissatisfied | |
|  |  |  |  |  |  | 3) Dissatisfied | |
| 5.4) | What support do you get from your family? | | | | | ……….. | |
| **Perceived health status** | | | | | | | |
| 6.1) | | How is your Health Status? | | 1) Good | | |  |
|  |  |  |  | 2) ok | | |  |
|  |  |  |  | 3) Bad | | |  |
| 6.2) | | Do you have any disease? | | 1) Yes | | | If, No go to 6.10 |
|  |  |  |  | 2) No | | |  |
| 6.3) | | What type of disease do you have? | | 1) Chronic | | |  |
|  |  |  |  | 2) Acute | | |  |
| 6.4) | | Do you have cataract? | | 1) Yes | | |  |
|  |  |  |  | 2) No | | |  |
| 6.5) | | Do you have diabetes? | | 1) Yes | | |  |
|  |  |  |  | 2) No | | |  |
| 6.6) | | Do you have CVD? | | 1) Yes | | |  |
|  |  |  |  | 2) No | | |  |
| 6.7) | | Do you have Bone related disease? | | 1) Yes | | |  |
|  |  |  |  | 2) No | | |  |
| 6.8) | | Do you have Kidney disease? | | 1) Yes | | |  |
|  |  |  |  | 2) No | | |  |
| 6.9) | | Do you have respiratory related disease? | | 1) Yes | | |  |
|  |  |  |  | 2) No | | |  |
| 6.10) | | Are you disabled? | | 1) Yes | | |  |
|  |  |  |  | 2) No | | |  |
| 6.11) | | Do you any mental problem? | | 1) Yes | | |  |
|  |  |  |  | 2) No | | |  |
| **Distance and means of transportation** | | | | | | | |
| 7.1) | | | How far is your health facility from your home? | | …… Minutes | | |
| 7.2) | | | How do you go to health facility? | | 1) By walk | | |
|  |  |  |  |  | 2) By carrying in Bamboo basket | | |
|  |  |  |  |  | 3) Bus | | |
|  |  |  |  |  | 4) others | | |

### APPENDIX : INFORMED CONSENT AND QUESTIONNAIRE (NEPALI VERSION)

**k'jf{~rnljZjljBfno**

**>L d]l8sn P08 6]lSgsnsn]h**

**e/tk'/–!@, uf]Gb|fË, lrtjg**

**“cg'dltkq”**

gd:sf/,

d]/f] gfd sdn kf}8]n xf] . d clxn] hg:jf:Yo -la= lk= Pr=_ rf}yf] jif{df>L d]l8sn P08 6]lSgsn sn]h e/tk'/,lrtjgdf cWoog/t 5' . o;} cWoogsf] lznlznfdf d}n] o; laxfbL ufpkflnsf df ^) aif{ jf ;f] eGbf a9Lsf j[4nfO{ **“lk|Eofn]G; / :jf:y ;]jf k|of]udf c;/ ug]{ tTjx?”** ;DalGw cEof;sf] af/]df cWoog ub}{5' . tkfO{af6 k|fKt;'rgf o; cWoogsf] nflu dxTjk"0f{ /xg]5g\ / cWoog k|of]hgsf nflu dfq k|of]u x'g]5g\ . tkfO{af6 k|fKt ;Dk"0f{ ;'rgfx¿ uf]Ko /flvg]5g\ . o; cGt/jftf{df ;xefuLx'g] gx'g] s"/f tkfO{sf] OR5fdf e/ k5{ / cGt/jftf{ s|ddf lrQ ga'´]df h'g;'s} ;dodf cl:jsf/ ug{ ;Sg'x'g]5 . tkfO{sf] ;xefuLtfsf] ck]Iff /fVb5' .

s] tkfO{ ;xefuLtf hgfpg OR5's x'g'x'G5 <

!_ 5' .

@_ 5}g .

cGt/jftf{lbg]sf] gfd:- ldlt:-

3/d'nLsf] gfd M

7]ufgf:-

cGt/jftf{lbg]sf] ;Dks{ g+=

cGt/jftf{lbg]sf] ;xL

;fIfLsf];xL:-

| **k\|=g+** | | **k\|Zgx¿** | | **k\|ltls\|of** | | **l:sk** |  |
| --- | --- | --- | --- | --- | --- | --- | --- |
| **!= ;fdflhs tyf hg;f+lvs ljj/0f** | | | | | | |  |
| !=!_ | | pd]/ | | ======================== | |  |  |
| !=@_ | | ln+u | | !_ k'?if | |  |  |
|  | |  | | @_ dlxnf | |  |  |
| !=#_ | | j}jflxs l:ytL s] 5 < | | !_ljjflxt | |  |  |
|  |  |  |  | @_ cljjflxt | |  |  |
|  |  |  |  | #_Psn | |  |  |
|  |  |  |  | $_;DaGB laR5]t | |  |  |
| !=$_ | | tkfO{ s'gwd{ dfGg' x'G5 < | | !_lxGb' | |  |  |
|  |  |  |  | @_af}4 | |  |  |
|  |  |  |  | #_lqmZrLog | |  |  |
|  |  |  |  | $_ O{:nfd | |  |  |
|  |  |  |  | %_cGo-v'nfpg'xf];\_ ============= | |  |  |
| !=%_ | | tkfO{sf] kl/jf/sf] ;+Vof: | | ============================= | |  |  |
|  |  |  |  |  |  |  |  |
| !=^_ | | tkfOsf] 5f]/f5f]/L stL 5g < | | !_ 5f]/f ===================== | |  |  |
|  |  |  |  | @_ 5f]/L ======================= | |  |  |
| !=&_ | | kl/jf/sf] k\|sf/: | | !_Psn | |  |  |
|  |  |  |  | @_ ;+o'Qm | |  |  |
|  |  |  |  | #_ a[xt | |  |  |
| !=*_ | | tkfO{sf] hft s] xf]< | | !_a\|fx\d0f | |  |  |
|  |  |  |  | @_ If]qL | |  |  |
|  |  |  |  | #_hghftL -v'nfpg]_ ============ | |  |  |
|  |  |  |  | $cflwafl; | |  |  |
|  |  |  |  | %_blnt | |  |  |
|  |  |  |  | ^_cGo-v'nfpg'xf];_ =============================== | |  |  |
| !=(_ | | tkfO{n] slt k9\g'' ePsf] 5 < | | !_lg/If/ @_sIff | |  |  |
| !=!)_ | | tkfOsf] k]zf s]yLof]] < | | !_ u[lx0fL | |  |  |
|  |  |  |  | @_ s[lif | |  |  |
|  |  |  |  | #_;]jf-hflu/_ $_>lds | |  |  |
|  |  |  |  | %_Aofkf/ | |  |  |
|  |  |  |  | ^_a}b]lzs/f]huf/ | |  |  |
|  |  |  |  | &_ a]/f]huf/ | |  |  |
|  |  |  |  | *_cGo-v'nfpg'xf];\_ ==============================\= | |  |  |
| !=!! | | tkfOsf] /f]huf/L sf] cj:yf s] 5 < | | !_ a]/f]huf/L | |  |  |
|  |  |  |  | @_ l/6fP8 | |  |  |
|  |  |  |  | #_ /f]huf/ | |  |  |
|  |  |  |  |  | |  |  |
| !=!@ | | tkfO{sf] kl/jf/sf] d'VocfDbfgLsf] ;\|f]t s] xf] < | | !_ u[lx0fL | |  |  |
|  |  |  |  | @_ s[lif | |  |  |
|  |  |  |  | #_;]jf-hflu/_ $_>lds | |  |  |
|  |  |  |  | %_Aofkf/ | |  |  |
|  |  |  |  | ^_a}b]lzs/f]huf/ | |  |  |
|  |  |  |  | =&_ a]/f]huf/ | |  |  |
|  |  |  |  | *_ cGo-v'nfpg'xf =============================\= | |  |  |
| !=!#_ | | tkfO{sf] kl/jf/sf] d'VocfDbfgLn] slt dlxgf ;Ddvfg k'U5 < | | !_ ^ dlxgfeGbfsd | |  |  |
|  |  |  |  | @_ ^–!@ dlxgf ;Dd | |  |  |
|  |  |  |  | #_ !@ dlxgfeGbf a9L | |  |  |
| !=!$_ | | tkfO{sf] kl/jf/sf] **jfifL{s** cfDbfgL] slt x'G5 < | | !_ ===================== | |  |  |
|  |  |  |  |  |  |  |  |
|  |  |  |  |  |  |  |  |
|  |  |  |  |  |  |  |  |
| !=!% | | tkfO{sf] JolQmut vr{ s;n] Aoxf]5{ < | | !_ cfkm} | |  |  |
|  |  |  |  | @_ kl/jf/ | |  |  |
|  |  |  |  | #_ gft]bf/ | |  |  |
|  |  |  |  | $_ ;fyLefO | |  |  |
|  |  |  |  | %_ cGo====================== | |  |  |
| !=!^ | | tkfO[{sf] s'g} w'dkfg lng] aflg 5 < | | !_ af/Daf/ | |  |  |
|  |  |  |  | @_ slxn]sfxL | |  |  |
|  |  |  |  | # vfP/ 5f]8]sf] | |  |  |
|  |  |  |  | $_ slxNo} vflbg | |  |  |
| !=!& | | tkfOn] s'g w'dkfg ug'{x'G5 < | | !_ l;u/]6 | |  |  |
|  |  |  |  | @_ t+afs' | |  |  |
|  |  |  |  | #_ ;'tL{ | |  |  |
|  |  |  |  | $_ kfg | |  |  |
|  |  |  |  | %_ cGo-v'nfpg'xf];\_ ============ | |  |  |
| !=!* | | tkfO[{sf] s'g} 'dwkfg lng] aflg 5 < | | !_ af/Daf/ | |  |  |
|  |  |  |  | @_ slxn]sfxL | |  |  |
|  |  |  |  | $_ vfP/ 5f]8]sf] | |  |  |
|  |  |  |  | #_ slxNo} vflbg | |  |  |
| **@ wg ;'rfGs** | | | | | | | |
| @=! | tkfO{sf] 3/sf] lkpg] kfgLsf] d'Vo >f]t s] xf]nf < | | ·        cfkm\g} 3/df /x]sf] kfOk af6 | |  | | |
|  |  |  | ·        l5d]sLsf] 3/sf] kfOk af6 | |  |  |  |
|  |  |  | ·        6\o'a j]n | |  |  |  |
|  |  |  | ·        Ogf/ | |  |  |  |
|  |  |  | ·        ;fj{hflgs wf/f | |  |  |  |
|  |  |  | ·        d'naf6 | |  |  |  |
| @=@ | tkfO{x?n] s:tf] lsl;dsf] rkL{ k\|of]u ug'{x'G5 < | | ·        cfw'lgs rkL{ | |  | | |
|  |  |  | ·        vfN8]rkL{ | |  |  |  |
| @=# | tkfO{sf] 3/df vfgf ksfpgsf nflu s] O{Gwg k\|of]u ul/G5 < | | ·        ljh'nL | |  | | |
|  |  |  | ·        UofF; | |  |  |  |
|  |  |  | ·        afof]UofF; | |  |  |  |
|  |  |  | ·        dl§t]n | |  |  |  |
|  |  |  | ·        sf]O{nf÷bfp/f | |  |  |  |
|  |  |  | ·        u'O{7f | |  |  |  |
|  |  |  | ·        cGo -pNn]v ug'{xf];\_ ============================ | |  |  |  |
|  |  |  |  | |  |  |  |
| @=$ | tkfO{sf]3/df eF'O{ s] n] ag]sf]5 < | | ·        df6f]÷jfn'jf÷ uf]j/ | |  | | |
|  |  |  | ·        afF;÷v/ | |  |  |  |
|  |  |  | ·        sf7 laR5\ofPsf] | |  |  |  |
|  |  |  | ·        6fon | |  |  |  |
|  |  |  | ·        l;d]G6 | |  |  |  |
|  |  |  | ·        cGo -pNn]v ug'{xf];\_ ============================ | |  |  |  |
| @=% | tkfO{sf]3/df 5fgf s] n] ag]sf] 5< -**d'Vo j:t'**_ | | ·        afF; | |  | | |
|  |  |  | ·        sf7 | |  |  |  |
|  |  |  | ·        v/ | |  |  |  |
|  |  |  | ·        l6g | |  |  |  |
|  |  |  | ·        6fOn | |  |  |  |
|  |  |  | ·        l;d]G6 | |  |  |  |
|  |  |  | ·        cGo -pNn]v ug'{xf];\_ ============================ | |  |  |  |
| @=^ | tkfO{sf] 3/df leQf s] n] ag]sf] 5 < | | ·        df6f] / sf7 | |  | | |
|  |  |  | ·        af; /df6f] | |  |  |  |
|  |  |  | ·        9'Ëuf /df6f] | |  |  |  |
|  |  |  | ·        KnfO{ | |  |  |  |
|  |  |  | ·        l;d]G6 Ans | |  |  |  |
|  |  |  | ·        O§f | |  |  |  |
| @=& | s] tkfO{sf] 3/df lgDg ;'ljwfx? 5g\ < | | ·        lah'nL | |  | | |
|  |  | | ·        /]l8of] | |  |  |  |
|  | -Ps jf Ps eGbf al9 pQ/_ | | ·        l6=eL= | |  |  |  |
|  |  | | ·        df]afO{n kmf]g | |  |  |  |
|  |  | | ·        cGo kmf]g | |  |  |  |
|  |  | | ·        lk\|mh | |  |  |  |
|  |  | | ·        sDKo"6/ | |  |  |  |
|  |  | | ·        38L | |  |  |  |
|  |  | | ·        cGo-pNn]v ug'{xf];\ _============================ | |  |  |  |
| @=* | s_ tkfO{sf] 3/df ;jf/Lsf lghL ;fwgx? 5g < | | !=5g\ | | olb 5}g eg] | | |
|  |  |  | )=5}gg\ | | k\|=g= @=!) dfhfg] | | |
| @=( | v_ olb 5g eg]s'g s'g 5g< | | ·        ;fO{sn÷l/S;f | |  | | |
|  |  |  | ·        df]6/;fO{sn÷:s'6/ | |  |  |  |
|  |  |  | ·        6]Dkf] | |  |  |  |
|  |  |  | ·        sf/ jf 6«s | |  |  |  |
|  |  |  | ·        cGo -pNn]v ug'{xf];\ _============================ | |  |  |  |
| @=!) | tkfO{sf] 3/df s;}sf] gfddf v]lt x'g;Sg] hUuf 5 < | | != 5 | |  | | |
|  |  |  | )= 5}g | |  |  |  |

| **# :jf:y ;]jf x]/rfxsf] k\|of]u** | | | | | | |  |
| --- | --- | --- | --- | --- | --- | --- | --- |
| #=! | | xh'/n] uPsf] ! aif{df :jf:y ;]jf k\|of]u ug'{ eof] < | ! u/] @ u/lg | |  | |  |
|  |  |  |  |  |  |  |  |
| **$ :jf:y ;]jf x]/rfx** | | | | | | | |
| $=! | xh'/ la/fdL kbf{ ;a}eGbf klxn] sxf+ hfg'x'G5 < | | | !_wfdL emfs\|L | |  | |
|  |  |  |  | @_ :jf:y rf}sL | |  |  |
|  |  |  |  | #_ k\|fylds :jf:y s]Gb\| | |  |  |
|  |  |  |  | $_ ;/sf/L c:ktfn | |  |  |
|  |  |  |  | % lghL c:ktfn | |  |  |
|  |  |  |  | ^_ cGo=========== | |  |  |
| $=@ | xh'/n] uPsf] ! jif{df slt k6s :jf:y ;]jf k\|of]u ug'{ eof] < | | | !_ =============== k6s | |  | |
| $=# | xh'/:jf:y ;:yfsf] ;]jf af6 ;Gt'i6 x'g"x'G5 < | | | !_ lgoldt r]s ug{  @_ :jf:y ;d:of eP/ | |  | |
| $=$ | xh'/:jf:y ;:yfsf] ;]jf af6 ;Gt'i6 x'g"x'G5 < | | | !_;Gt"i6  @_ gt ;Gt"i6 gt c;Gt"i6  #_ c;Gt"i6 | |  | |

| $=% | | tkfO :jf:y ;:yfsf] ;]jf lng stL ;do s'g'{ k5{ < | | !_nfdf] ;do | |  | |
| --- | --- | --- | --- | --- | --- | --- | --- |
|  |  |  |  | @_ l7s} | |  |  |
|  |  |  |  | #_ l56} x'G5 | |  |  |
| $=^ | | tkfO uPsf] :jf:y ;:yfdf sd{rf/L pknAw x'G5g\ < | | !_ ;w} x'G5g . | |  | |
|  |  |  |  | @ slxn]sfxL x'G5g\ . | |  |  |
|  |  |  |  | #_ slxNo} x'b}gg\ . | |  |  |
| $=& | | tkfO uPsf] :jf:y ;:yfdf cf}ifwL k\|ofKt dfqfdf pknAw x'G5g\ < | | !_ k\|ofKt 5g\ | |  | |
|  |  |  |  | @_ yf]/} 5g\ | |  |  |
|  |  |  |  | #_ 5b} 5}gg\ | |  |  |
| $=* | | tkfO k\|tL :jf:y sdL{sf] JoJfxf/ s:tf] lyof] < | | !_ /fd\|f] | |  | |
|  |  |  |  | #_ l7s} | |  |  |
|  |  |  |  | @_ g/fd\|f] | |  |  |
| $=( | | tkfOnfO{ :jf:y ;]jf lbbf gd\|tfsf ;fy af]N5g\ < | | !_ af]N5g | |  | |
|  |  |  |  | @_ af]Nb}Gg\ | |  |  |
| $=!) | | tkfOnfO{ :jf:y ;]jf lbg] sd{rf/Lsf] s'/f a'emfpg] Ifdtf s:tf] 5 < | | !_ /fd\|f] | |  | |
|  |  |  |  | #_ l7s} | |  |  |
|  |  |  |  | @_ g/fd\|f] | |  |  |
| $=!! | | tkfOnfO{ :jf:y sd{rf/n] hf+Rg stL ;do nufp5g\ < | | !_ ============= | |  | |
| $=!@ | | tkfOn] To+xf ePsf] :jf:y ;]jfsf] vr{ tkfO cfkm} ltg{ ;Sg'x'G5 | | !_ ;S5' | |  | |
|  |  |  |  | @_ ;lSbg | |  |  |
| $=!# | tkfOsf] :jf:y vr{sf] cfDbfgLsf] ;\|f]t s] xf] < | | !_ ladf | |  | |  |
|  |  |  | @_ etf | |  |  |  |
|  |  |  | #_ 5f]/f5f]/L af6 | |  |  |  |
|  |  |  | $_ k]G;g | |  |  |  |
|  |  |  | %cGo -v'nfpg'xf];_============ | |  |  |  |
| $=!$ | :jf:y ladf k\|of]u ug'{ ePsf] 5 < | | ! 5' | |  | |  |
|  |  |  | @ 5}g\. | |  |  |  |
| $=!% | :jf:y ljdfaf6 s]s] ;]jf kfpg'e5 < | |  | |  | |  |
| $=!^ | xh'/:jf:y ladf af6 kfOg] ;]jf af6 ;Gt'i6 x'g"x'G5 < | | !_ ;Gt"i6 | |  | |  |
|  |  |  | @_ gt ;Gt'i6 gt c;Gt'i6 | |  |  |  |
|  |  |  | #_ c;Gt'i6 | |  |  |  |
| $=!& | xh'/n]:jf:y ;:yfsf] ;a} ;]jf l:jsf/ ug'{ x'G5 < | | u5'{ | |  | |  |
|  |  |  | ulb{g | |  |  |  |
| $=!* | xh'/:jf:y ;:yf hfbf sf] ;u+ hfg'x'G5 < | | !_ cfkm}+ | |  | |  |
|  |  |  | @_ l>dfg | |  |  |  |
|  |  |  | #_ l>dtL | |  |  |  |
|  |  |  | $_ 5f]/f | |  |  |  |
|  |  |  | % 5f]/L ^ cGo v'nfp_===================== | |  |  |  |

| **% kl/jf/ af6 ;xfotf** | | | | | | |  |
| --- | --- | --- | --- | --- | --- | --- | --- |
| %=! | tkfO{sf] kl/jf/df :jf:y ;]jf lng] ;DaGwdf s;n] lg0f{o u5{ < | | | !_ cfkm}+ | |  |  |
|  |  |  |  | @_ l>dfg | |  |  |
|  |  |  |  | #_ l>dtL | |  |  |
|  |  |  |  | $_ 5f]/f | |  |  |
|  |  |  |  | %_ 5f]/L | |  |  |
|  |  |  |  | ^_cGo – v'nfp_========= | |  |  |
| %=@ | tkfOnfO kl/jf/n] :jf:y ;]jf lng] ;DaGwdf ;xof]u ug'{x'G5 < | | | !_ ug'{x'G5 | |  |  |
|  |  |  |  | @_ ug'{x'b}g | | olb ug'{x'b}g eg]k\|=g=^=! dfhfg] |  |
| %=# | s] tkfO kl/jf/sf] ;xof]u af6 ;Gt'i6 x'g'x'G5 < | | | !_;Gt"i6 | |  |  |
|  |  |  |  | @_ gt ;Gt"i6 gt c;Gt"i6 | |  |  |
|  |  |  |  | #_ c;Gt"i6 | |  |  |
| %=$ | tkfOnfO kl/jf/af6 s]s] ;xof]u ug'{x'G5 < | | | =========== | |  |  |
| **^ :jf:y cj:yf** | | | | | | | |
| ^=! | | tkfOsf] :jf:y cj:yf s:tf] 5 < | !_ /fd\|f] | |  | | |
|  |  |  | @_ l7s} | |  |  |  |
|  |  |  | #_ g/fd\|f] | |  |  |  |
| [^=@](mailto:%5E=@) | | tkfOnfO s'g} /f]u nfu]sf] 5 < | !_ 5 | | olb 5}g eg] | | |
|  |  |  | @_ 5}g | | k\|=g=%=% dfhfg] | | |
| ^=# | | tkfOnfO s'g /f]u nfu]sf] 5 < | !_ lb3{sflng | |  | | |
|  |  |  | @_ ;fdfGo | |  |  |  |
| ^=$ | | tkfOnfO df]tL laGb' /f]u nfu]sf] 5 < | ! 5 @ 5}g | |  | | |
| ^=% | | tkfOnfO;'u//f]u nfu]sf] 5 < | ! 5 @ 5}g | |  | | |
| ^=^ | | tkfOnfO d'6" ;daGwL /f]u nfu]sf] 5 < | ! 5 @ 5}g | |  | | |
| ^=& | | tkfOnfO x8\8L ;daGwL /f]u nfu]sf] 5 < | ! 5 @ 5}g | |  | | |
| ^=* | | tkfOnfO ls8\gL ;DaGwL /f]u nfu]sf] 5 < | ! 5 @ 5}g | |  | | |
| ^=( | | tkfOnfO Zjfzk\|Zjfz ;daGwL /f]u nfu]sf] 5 < | ! 5 @ 5}g | |  | | |
| ^=!) | | +tkfOdf s'g} ckf+utf 5 < | ! 5 @ 5}g | |  | | |
| ^=!! | | tkfOnfO dfgl;s ;d:of 5 < | ! 5 @ 5}g | |  | | |

| **& b'/L / oftfoftsf] ;fwg** | | | |
| --- | --- | --- | --- |
| &=! | tkfOsf] 3/af6 :jf:y ;:+yf stL 6f9f 5 < | ============= ldg]6 |  |
