# Supplementary material for: Health care service utilization among elderly in rural setting of Gandaki province, Nepal: a mixed method study
Source: Front Health Serv. 2024 Sep 25;4:1321020. doi: 10.3389/frhs.2024.1321020 (PMC11462626; doi:10.3389/frhs.2024.1321020)
Supplement: Supplementary file 4 [file Table4.docx]

**Appendix. 4. QUALITATIVE DATA COLLECTION TOOL (In-depth interview guide)**

**Rural Municipality:**

**Ward No.:**

**Tole:**

**Venue of interview:**

**Interviewee:**

**Interviewer:**

**Date of interview:**

**Background Characteristics**

| Age | Sex | Education | Occupation | Caste | Source of income | Family type | No. of sons/daughter | Living alone/with children/spouse | Distance to health facility | visited health service  in last one year | Reason for using the health service |
| --- | --- | --- | --- | --- | --- | --- | --- | --- | --- | --- | --- |
|  |  |  |  |  |  |  |  |  |  |  |  |

1. How is your health status? How?

2. How frequently do you use health service of health facilities? Where do you visit for health service?

3. Do you have any problem in using health service of the health facilities? (Explore-**WHAT**/**WHY?** Individual, Family, Community, Health service related factors)

4. How is the support of your family for you using health service? Is it sufficient? (Explore- What/Why/How?)

5. How difficult is it to travel to health facility? (Explore- Why?)

6. Is there any problem to afford health services? (Explore- What/Why?)

7. Are you in Health Insurance Scheme? How has it helped to access health service?

7. How do you find the services of health facilities? (Explore- Waiting time, Checkup, Behavior of service providers, Cost of service, other arrangements, etc.)

8. In your opinion, what is required for elderly people to use health service sufficiently whenever they need?

(Explore:

- Individual factors,

- Support from Family,

- Community factors,

- Health Service related factors

**गुणात्मक डाटा सङ्कलन उपकरण (गहिरो अन्तर्वार्ता गाइड)**

गाउँपालिका:

वडा नम्बर:

टोल:

अन्तर्वार्ता स्थल:

साक्षात्कारकर्ता:

साक्षात्कारकर्ता:

अन्तर्वार्ताको मिति:

पृष्ठभूमि विशेषताहरू

| pd]/ | लैङ्गिक | शिक्षा | पेशा | जात | आयको स्रोत | परिवारको प्रकार | छोरा/छोरी एक्लै बस्ने/बच्चा/पति/पत्नीसँग | स्वास्थ्य सुविधाको दुरी भ्रमण गरिएको स्वास्थ्य सेवा | पछिल्लो एक वर्षमा स्वास्थ्य सेवा प्रयोग गर्नुको कारण |
| --- | --- | --- | --- | --- | --- | --- | --- | --- | --- |
|  |  |  |  |  |  |  |  |  |  |

!. तपाईको स्वास्थ्य अवस्था कस्तो छ? कसरी?

२. तपाई स्वास्थ्य संस्थाको स्वास्थ्य सेवा कति पटक प्रयोग गर्नुहुन्छ? स्वास्थ्य सेवा लिन कहाँ जानुहुन्छ ?

३. स्वास्थ्य संस्थाको स्वास्थ्य सेवा प्रयोग गर्न तपाईलाई कुनै समस्या छ ? (खोज्नुहोस्-के/किन? व्यक्ति, परिवार, समुदाय, स्वास्थ्य सेवा सम्बन्धित कारकहरू)

$=स्वास्थ्य सेवा प्रयोग गर्दा तपाईंको परिवारको सहयोग कस्तो छ? के यो पर्याप्त छ? (अन्वेषण- के/किन/कसरी?)

%. स्वास्थ्य संस्थामा यात्रा गर्न कत्तिको गाह्रो छ? (अन्वेषण- किन?)

^. स्वास्थ्य सेवा किन्न कुनै समस्या छ? (अन्वेषण- के/किन?)

&. के तपाई स्वास्थ्य बीमा योजनामा ​​हुनुहुन्छ? यसले स्वास्थ्य सेवाको पहुँचमा कसरी मद्दत गरेको छ ?

*. स्वास्थ्य संस्थाका सेवाहरू कसरी प्राप्त गर्नुहुन्छ? (अन्वेषण गर्नुहोस्- पर्खने समय, जाँच, सेवा प्रदायकहरूको व्यवहार, सेवाको लागत, अन्य व्यवस्थाहरू, आदि)

(. तपाईको विचारमा वृद्धवृद्धाहरूले आवश्यक परेको बेला स्वास्थ्य सेवाको पर्याप्त प्रयोग गर्न के गर्नुपर्छ?

(अन्वेषण गर्नुहोस्:

- व्यक्तिगत कारकहरू,

- परिवारबाट सहयोग,

- समुदाय
