# Supplementary material for: Health care service utilization among elderly in rural setting of Gandaki province, Nepal: a mixed method study
Source: Front Health Serv. 2024 Sep 25;4:1321020. doi: 10.3389/frhs.2024.1321020 (PMC11462626; doi:10.3389/frhs.2024.1321020)
Supplement: Supplementary file 5 [file Table5.docx]

Appendix 5. Identified theme and subtheme during the analysis

| **Themes** | **Sub-themes** |
| --- | --- |
| **Health status of elderly** | Neither Good  Nor Bad |
| **Problem arises during utilization of health care services** | Personal Problem  Family Problem  Community Problem  Health Facility |
| **Support from family** | Support from Family Members  No Support from Family Members |
| **Health services** | Waiting time to access health care services.  Time to checkup  Afford to get health care services  Utilization of Health insurances.  Attitudes of health staff towards elderly people.  Attitudes of health staff towards elderly people.  Elderly perception on quality health care services |
| **Requiring needs to use health care services for elderly people** | Individual  Family  Community  Health facility |
