# Supplementary material for: Health care service utilization among elderly in rural setting of Gandaki province, Nepal: a mixed method study
Source: Front Health Serv. 2024 Sep 25;4:1321020. doi: 10.3389/frhs.2024.1321020 (PMC11462626; doi:10.3389/frhs.2024.1321020)
Supplement: Supplementary file 6 [file Table6.docx]

Appendix 6. sociodemographic characteristics of elderly for qualitative (n=18)

| **Characteristics** | **Frequency** | **Percentage** |
| --- | --- | --- |
| **Age** | | |
| 60-69 | 6 | 33.3 |
| 70-79 | 9 | 50 |
| 80-89 | 3 | 16.7 |
| **Average Years** | 73.11 | |
| **Sex** | | |
| Male | 8 | 44.4 |
| Female | 10 | 55.6 |
| **Education** | | |
| Illiterate | 12 | 66.7 |
| Literate | 6 | 33.3 |
| **Occupation** | | |
| Agriculture | 15 | 83.3 |
| Services | 3 | 16.7 |

| **Caste** | | |
| --- | --- | --- |
| Brahmin/Chhetri | 5 | 27.8 |
| Advantage Janajaati | 2 | 11.1 |
| Dalit | 11 | 61.1 |
| **Family type** | | |
| Nuclear | 12 | 66.7 |
| Joint | 6 | 33.3 |
| **Distance** | | |
| Less than 30 min. | 15 | 83.3 |
| 30 0r more than 30 | 3 | 16.7 |
| **Frequency of health facility visited** | | |
| 1 times | 2 | 16.7 |
| 2 times | 4 | 33.3 |
| 3 or more than 3 | 6 | 49 |
| **Reason for visit health facility** | | |
| Regular checkup | 5 | 41.7 |
| Health problem | 7 | 58.3 |
| **Utilization of health services** | | |
| No | 6 | 33.33 |
| Yes | 12 | 66.67 |
