# Supplementary material for: Health care service utilization among elderly in rural setting of Gandaki province, Nepal: a mixed method study
Source: Front Health Serv. 2024 Sep 25;4:1321020. doi: 10.3389/frhs.2024.1321020 (PMC11462626; doi:10.3389/frhs.2024.1321020)
Supplement: Supplementary file 7 [file Table7.docx]

**Appendix- S7. Appendix 7.** Relationship with different factors for health service utilization.

| **Characteristics** | **Crude Odds Ratio** | **Model I**  **Socio-Demographic** | **Model II**  **Health Service** | **Model III**  **Socio-Demographic and Health service** | **Model IV**  **Forward Stepwise LR** |
| --- | --- | --- | --- | --- | --- |
|  | cOR | aOR(95 %CI) | | | |
| **Age** | Ref (60-69 Years) | | | | |
| 70-79 | 0.636(0.386-1.401) | 0.412(0.191-0.857) |  | 0.537(0.21-1.326) |  |
| ≥80 | 0.795-0.429-1.486) | 0.579(0.248-1.343) |  | 0.827(0.263-2.635) |  |
| **Sex** | Ref (Male) | | | | |
| Female | 1.260(0.813-1.957) | 0.971(0.496-1.902) |  | 0.636(0.255-1.537) |  |
| **Ethnicity** | Ref(Brahmin/Chhetri) | | | | |
| Advantaged Janajati | **2.804(1.289-6.808)** | 3.549(1.273-11.855) |  | **3.728(1.062-15.887)** |  |
| Disadvantaged Janajati | 0.881(0.50-1.567) | 0.774(0.346-1.753) |  | 0.457(0.165-1.254) |  |
| Dalit | 0.852(0.478-1.529) | 0.772(0.33-1.841) |  | 0.733(0.243-2.282) |  |
| **Family Type** | Nuclear (Ref) | | | | |
| Joint | 0.963(0.622-1.493) | 0.512(0.269-0.953) |  | 0.852(0.359-2.005) |  |
| **Education** | Basic (Ref) | | | | |
| Secondary | 1.009(0.633-1.622) | 1.097(0.522-2.347) |  | 0.683(0.246-1.906) |  |
| **Employment Status** | Unemployed (Ref) | | | | |
| Employed | 0.842(0.411-1.787) | 0.677(0.237-2.056) |  | 1.854(0.427-9.255) |  |
| **Decision to take Health Service in family** | Self(Ref) | | | | |
| Spouse | 0.801(0.408-1.600) | 0.772(0.29-2.15) |  | 0.74(0.214-2.704) |  |
| Children and their spouse | 1.462(0.868-2.512) | 0.892(0.449-1.773) |  | 0.620(0.252-1.500) |  |
| **Satisfaction from family support** | Satisfied (Ref) | | | | |
| Neither satisfied nor dissatisfied | 0.732(0.390-1.401) | 0.955(0.445-2.117) |  | 1.212(0.456-3.413) |  |
| Dissatisfied | **0.440(0.266-0.727)** | 1.241(0.155-26.128) |  | 1.301(0.106-37.167) |  |
| **Type of support by family member** | Financial Support(Ref) | | | | |
| Looking after parents | 1.886(0.734-5.183) | 2.211(0.761-6.873) |  | 1.344(0.373-5.063) |  |
| Support in maintaining personal hygiene | 1.565(0.636-4.013) | 1.747(0.653-4.871) |  | 1.363(0.389-4.955) |  |
| Support in food | 1.583(0.552-5.001) | 1.18(0.353-4.255) |  | 1.062(0.206-5.779) |  |
| Support in treatment | 1.408(0.676-2.917) | 1.395(0.616-3.151) |  | 1.257(0.428-3.639) |  |
| Help in farming | 1.211(0.406-3.919) | 1.431(0.416-5.301) |  | 1.284(0.269-6.611) |  |
| **Wealth Index** | Rich (Ref) | | | | |
| Medium | 0.873(0.510-1.491) | 0.566(0.262-1.188) |  | 0.401(0.138-1.077) | 0.669(0.348-1.269) |
| Poor | 0.927(0.540-1.590) | 0.429(0.19-0.938) |  | 0.178(0.057-0.51) | 0.602(0.314-1.133) |
| **Nearest health facility** | Health Post (Ref) | | | | |
| Government hospital | 0.653(0.311-1.375) |  | 0.111(0.031-0.345) | 0.046(0.009-0.193) | 0.294(0.125-0.686) |
| Private clinic | **1.864(1.133-3.123)** |  | 0.781(0.41-1.483) | 0.503(0.202-1.241) | 1.025(0.575-1.837) |
| **Satisfied with available health care services** | Satisfied (Ref) | | | | |
| Dissatisfied | **0.253(0.157-0.402)** |  | 0.394(0.216-0.713) | 0.488(0.214-1.091) |  |
| **Availability of Health Staffs** | Always (Ref) | | | | |
| Not always | **4.165(2.186-8.196)** |  | 0.359(0.192-0.663) | 0.375(0.147-0.931) |  |
| **Availability of medicine** | Sufficient (Ref) | | | | |
| Not sufficient | **0.400(0.234-0.674)** |  | 0.534(0.268-1.052) | 0.372(0.143-0.924) | 0.34(0.19-0.592) |
| Not available | **0.365(0.138-0.085)** |  | 0.055(0.018-0.153) | 0.014(0.002-0.068) | 0.026(0.009-0.066) |
| **Attitude of health staffs** | Good (Ref) | | | | |
| Not good | **0.454(0.290-0.707)** |  | 1.599(0.829-3.176) | **2.943(1.15-8.046)** |  |
| **Able to afford health care service** | No (Ref) | | | | |
| Yes | **2.195(1.360-3.547)** |  | 1.573(0.855-2.875) | 1.94(0.797-4.793) |  |
| **Means of Transport** | On foot (Ref) | | | | |
| Bus | **2.676(1.148-7.324)** |  | 3.455(0.884-16.55) | **8.397(1.587-55.091)** |  |
| **Health Status** | Good (Ref) | | | | |
| Bad | **2.227(1.431-3.491)** |  | 1.922(1.106-3.364) | 0.861(0.375-1.924) |  |
| **Distance to Health Facility** | 0-15 minutes (Ref) | | | | |
| 16-30 minutes | **0.453(0.271-0.751)** |  | 0.407(0.209-0.776) | 0.658(0.256-1.669) |  |
| >30 minutes | 0.750(0.423-1.341) |  | 0.845(0.373-1.948) | 0.926(0.299-2.983) |  |
| *** Akaike Information Criterion (AIC)** | **-** | **325.26** | **353.36** | **273.1** | **383.84** |

*Model I- Socio-demographic, Model II- Health Service, Model III- Socio-demographic and Health Services, Model IV- Stepwise Logistic*
